# Dr. Stephen G. Warren

**Published:** 1913-11

**Authors:** 


Dr. Stephen G. Warren, Cleveland Homeopathic, 1865, died at his home in Attica in October. He was born in Portmen, O., in 1840.

